# Critical Appraisal of Qualitative Studies of Muslim Females’ Perceptions of Physical Activity Barriers and Facilitators

**DOI:** 10.3390/ijerph16245040

**Published:** 2019-12-11

**Authors:** David Kahan

**Affiliations:** School of Exercise and Nutritional Sciences, San Diego State University, ENS Building 315, 5500 Campanile Drive, San Diego, CA 92182-7251, USA; dkahan@sdsu.edu

**Keywords:** focus group interviews, methodological rigor, reporting transparency, religion

## Abstract

Muslim women’s perceptions of cultural, religious, and secular determinants of physical activity have been studied for many years, with information typically acquired through focus groups or interviews. Multiple reviews synthesizing the research have been published, however, individual studies have not been scrutinized for their quality/rigor. Therefore, I critically appraised the quality of the body of qualitative research studies that utilized focus groups to identify Muslim women’s perceptions of physical activity barriers and facilitators. I utilized 26 items from the Consolidated Criteria for Reporting Qualitative Research (COREQ) to assess the quality of 56 papers published between 1987 and 2016. Using crosstabulations, I also examined associations between paper quality (low vs. high) and binary categorical variables for impact factor, maximum paper length allowed, publication year, and database the paper was indexed. Overall, papers averaged only 10.5 of 26 COREQ reporting criteria and only two out of 26 items were reported in more than 75% of the papers. Paper quality was not associated with impact factor and length. High quality papers were more likely published more recently (i.e., 2011 or later) and in journals indexed in the PubMed database compared to low quality papers. There is contention among qualitative researchers about standardizing reporting criteria, and while the trend in quality appears to be improving, journal reviewers and editors ought to hold authors to greater accountability in reporting.

## 1. Introduction

In 2015, the global Muslim population numbered 1.8 billion persons and comprised 24.1% of the world’s population [1]. Exegesis of Islam’s guiding holy scripture, the Qu’ran, identifies more than two dozen verses that direct/support adherents’ health behavior including physical activity [2]. Hadith are a collection of traditional accounts and sayings of the Prophet Mohammed’s daily life that are separate from the Qu’ran and further guide adherents’ behavior. They contain references to multiple physical activities that Mohammed engaged in or supported including archery, horseback riding, running, swimming and wrestling [3]. Yet, in contemporary times, the prevalence of physical inactivity among Muslims is high and concerning. Specifically, 32.3% of Muslims residing in 38 Islamic countries were physically inactive with Muslim vs. non-Muslim countries being 1.2 times more likely physically inactive [4]. Muslim subpopulations particularly vulnerable to physical inactivity, relative to their counterparts, include Arabs (43.7%) and females (35.5%) [4]. 

Migration from Muslim-majority to non-Muslim-majority countries has occurred for centuries. Muslims living in the West in Australia, the United States, and European Union countries, for example, reflect a mélange of established multi-generation families, immigrants, asylum seekers, and refugees. Research into these peoples’ physical activity levels and behavior has accelerated over the last 20 years with an aim toward understanding the barriers to and facilitators of physical activity they encounter. Research participants perceive that barriers and facilitators to physical activity differ between the countries they currently reside and their autochthonous homelands [5,6,7]. Findings from these and other studies could inform the development of culturally and religiously tailored interventions for increasing physical activity, particularly in western, high human development countries that embrace cultural and religious pluralism and have the resources to accommodate the needs of a diverse population.

To date, published primary studies on physical activity barriers and facilitators among Muslims living in western societies have allowed for publication of multiple review articles. The reviews have collectively (1) subsumed Muslim populations under a smaller or larger population demographic (e.g., “ethnic minority groups,” “South Asian,” “culturally and linguistically diverse migrant groups”), (2) situated them within a smaller geographic sphere (e.g., United Kingdom, western Europe, western society), and/or (3) focused more narrowly on population segments (e.g., older adults, girls) [8,9,10,11,12,13,14,15,16]. Thus, generalization to the pan-Islamic population residing across western societies is limited. I was particularly interested in summarizing how Muslim females living in Western societies experience barriers to and facilitators of physical activity. Published physical activity interventions delivered to Muslim females have tapped into multiple layers of the socioecological model to redress intrapersonal (e.g., lack of self-efficacy, motivation, and knowledge), interpersonal (lack of social support), and environmental (i.e., lack of low-cost venues that comply with cultural/religious beliefs about women’s participation relative to modest dress and intermingling of the sexes) barriers [17,18,19]. Yet, to date, no synthesis of barriers to and facilitators of physical activity among this subpopulation has been published. I therefore set out to conduct such a review and in the process was surprised by the quality of eligible studies, which is the focus of the present paper.

Identifying physical activity barriers and facilitators entails interpretation of participants’ perceptions, which are particularly well suited for qualitative research [20]. Qualitative research methods such as in-depth and focus group interviews are considered valuable tools for understanding “the perceptions, beliefs, and values of a group’s participants and [are] particularly well suited to addressing cultural characteristics that impact on a population’s health” [21], p. 91 Qualitative research is focused on the human experience and condition [20]; focus groups foster social interaction and attempt to generate consensus about phenomena and thus may be particularly suitable for studies of Muslims, who are considered to more strongly espouse a collectivist ethos [22]. Muslim women (the group of interest in this study) share kinship as they navigate gender differentiation and patriarchy in Islamic society [23], and focus group interviews may be quite salient for understanding their perceptions of physical activity barriers and facilitators as Muslims overall, as well as those particular to their experience as Muslim women. 

The application of findings from this line of inquiry to clinical settings should ultimately increase Muslim women’s engagement in physical activity. Research interventionists and practitioners rely on qualitative research to be rigorous (i.e., high quality) if programs they develop based on research findings are to succeed. Concern has been expressed for various types of rigor (e.g., procedural, interpretive, evaluative) in the conduct and reporting of qualitative research in the health sciences [24]. Mays and Pope [25] contended that debate has centered on whether qualitative research should be held to the same quality standards as quantitative research and whether a unified view of quality can capture the various methods/models of qualitative inquiry (e.g., phenomenology, grounded theory, ethnography). Meanwhile, Tong et al. [26] consolidated items from 22 checklists for explicit and comprehensive reporting of qualitative studies of in-depth interviews and focus groups to derive the 32-item consolidated criteria for reporting qualitative research (COREQ). The COREQ is an example of a reporting guideline: The Enhancing the Quality and Transparency of Health Research (EQUATOR) Network is a repository of such guidelines and promotes their use by journal editors and publishers. A Delphi panel study of experts in qualitative research found near unanimous endorsement of some form of generalized reporting guideline to potentially increase “quality, rigor, and credibility of qualitative research” [27], p. 13.

The purpose of the present study was to evaluate the quality of focus group qualitative research on physical activity barriers and facilitators conducted with Muslim females living in non-Muslim, high human development countries. Quality was operationalized to reflect the degree to which studies reported items from the COREQ reporting guidelines. Secondarily, associations between various publication attributes and the quality of studies were explored.

## 2. Materials and Methods

### 2.1. Data Sources and Search Strategy

A literature search was conducted using 6 bibliographic databases: CINAHL, ERIC, PsycINFO, SPORTDiscus (all subsumed under EBSCO), PubMed, and Web of Science from earliest date through June, 2016. I used the following search string in PubMed and accordingly refined parameters for EBSCO and Web of Science databases to most broadly identify studies that might identify barriers and facilitators of physical activity encountered by Muslim women: 

(Somali * OR Islam * OR “South Asian” OR Arab * OR Somalia[mh] OR Islam[mh] OR Arabs[mh] OR Middle East[mh] OR Asia, Western[mh]) AND (women OR girl OR female OR gender OR female[mh] OR women[mh]) AND (sports OR exercise OR “physical activity” OR “physical education” OR walking OR swimming OR running OR soccer OR danc * OR motor activity[mh] OR physical education and training[mh] OR sports[mh]) AND (qualitative OR “focus group” OR interview OR qualitative research[mh] OR interviews as topic[mh] OR focus groups[mh]) 

Bibliographies of included studies were hand searched to identify additional papers that met eligibility criteria. 

### 2.2. Inclusion and Exclusion Criteria

**Figure 1:**
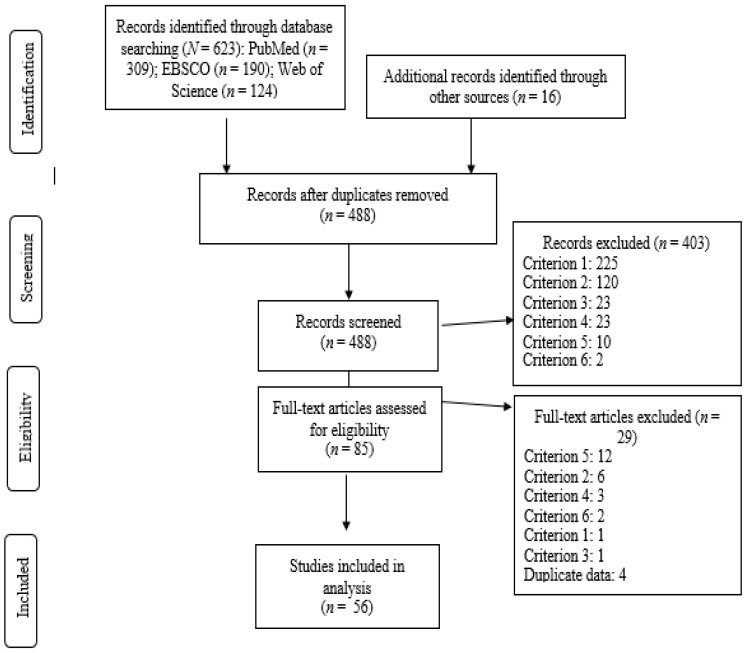
PRISMA flow diagram of search results and derivation of sample of qualitative studies.

Article eligibility criteria were based on a 6-level hierarchy that was subsequently applied during screening and eligibility stages of review (Figure 1): 

(1) country (non-Islamic majority countries classified by the United Nations Development Programme [28] as very high human development and whose Muslim minority population numbered ≥25,000 persons and comprised ≥ 0.5% of its 2010 population [29]; 

(2) non-therapeutic physical activity including exercise, physical education, and/or sport as a focus and mention of barriers or facilitators; 

(3) healthy populations including overweight; 

(4) qualitative or mixed-methods studies that utilized interview techniques (excluding reviews); 

(5) participants of any age identifiable as Muslim girls or women (excluding parents and providers); and

(6) non-interventions (including articles where pertinent data were collected prior to administration of an intervention); 

Intra-rater reliability four months post initial review was 98.1% agreement and κ = 0.88 (95% CI, 0.80–0.96). Concurrent interrater reliability between the author and trained academic colleague was 98.5% agreement and κ = 0.91 (95% CI, 0.85–0.98). Disagreements were discussed until consensus was reached.

### 2.3. Critical Ratings of Quality of Papers

Data addressing 24 of 32 items reported in the COREQ [26] were extracted from papers for independent (author and trained graduate assistant) coding and derivation of quality scores for papers. Two COREQ items were subdivided resulting in a 26-item checklist. Original COREQ items 1–5 (Domain 1: Research team and reflexivity—Personal Characteristics) and 30–32 (Domain 3: Analysis and findings—Reporting) were omitted from the checklist. Each item was coded 0 (absent), 0.5 (partially present), or 1 (fully present).

The median value of all papers’ quality scores (i.e., sum of the coded values across the 26 items) were used to differentiate between high- and low-quality papers. Interrater reliability was 83.9% agreement and κ = 0.68 (95% CI, 0.49–0.87). Further examination of the disagreements revealed that for 6 of the 9 disagreements the other rater’s quality score matched or was 0.5 point below the median quality score, which resulted in a study being classified “low quality” versus the other coder’s quality score being above the median quality score (i.e., high quality). When these 6 discrepancies were resolved only three studies’ quality scores remained classification disagreements (i.e., 94.6% agreement; κ = 0.89 (95% CI, 0.78–1.00)).

### 2.4. Analysis

I first conducted descriptive analyses (mean ± *SD*) based on each paper’s proportion of the maximum score of 26 achieved (i.e., across COREQ items) and for each COREQ item across papers (i.e., proportion of the maximum score of 56 achieved).

Four attributes (variables) were then developed to characterize each paper. These attributes were based on studies and commentaries that suggest: (1) the appearance of qualitative research in medical journals has increased since the late 1990s [30], (2) “the content of [a qualitative] article is often at the mercy of journal format, page length restrictions, and journal reviewers” [20] p. 133, and (3) qualitative studies are infrequently published in top ranked general medical, and health services and policy research journals and more frequently appear in low impact factor clinical journals [31,32]. 

First, the maximum length allowed in words for submissions to the published papers’ journals was obtained from journal websites’ posted instructions to authors. Three papers’ journals did not list this datum and a fourth’s journal had ceased publication in 2002 and no longer had a website. Two additional papers’ journals explicitly stated there were no restrictions on length and their length was tallied as the longest allowed word count across journals +1 word (i.e., 50,001). Six papers’ journals identified a maximum page length and these values were converted to word counts using an online app (https://wordcounter.net/words-per-page). The resulting median value of 6885 words differentiated shorter from longer paper length limits.

Second, the database in which a paper was found was distinguished. Papers found in PubMed—whether found only in that database or in it and another—were differentiated from papers found in Web of Science, EBSCO, or both. Thirty papers were found in PubMed while 26 papers were found in other databases.

Third, the quality of the journals in which papers were published was identified. I used SCImago [33], which ranks journals based on their SJR2 indicator, which was “designed to weight the citations according to the prestige of the citing journal, also taking into account the thematic closeness of the citing and the cited journals” [34] p. 675. Specifically, I extracted the highest quartile ranking across subject categories for a journal 2 years after a paper’s publication. Six papers were published in years that predated the first SCImago rankings in 1999 and/or were published in journals not listed in the SCImago database and thus could not be coded. Overall, 32, 16, and 2 papers were published in first, second, and third quartile-ranked journals, respectively. For analysis, journal quality was dichotomized into high (first quartile) and low (second and third quartiles).

Fourth, the median date of publication (i.e., 2011) was identified and used to dichotomize papers into older vs. newer publications.

Finally, I conducted crosstabulations, chi-square, and post-hoc odds ratios to explore relationships between these four attributes and the quality of papers (i.e., low vs. high).

### 2.5. Ethical Statements

This study did not involve human participants. Thus, given the nature of the research, the study was exempt from review by the university’s institutional review board. 

## 3. Results

### 3.1. Study Characteristics

From the 1262 initial search results, 56 studies published between 1987 and 2016 satisfied the inclusion criteria (Figure 1). Papers were published in 38 different journals with *Sport*, *Education & Society* publishing the most (9). The studies represented at least (i.e., not all studies reported sample size) 1036 Muslim female participants between the ages of five and 73-years-old residing in: The United Kingdom (21), United States (10), Australia (9), Norway (5), Canada (3), Denmark (2), Israel (2), Sweden (2), Belgium (1), and New Zealand (1). The women represented at least 30 national or ethnic groups (i.e., not all studies reported) with Pakistani (21), Somali (17), and Bangladeshi (12) most commonly represented across studies.

### 3.2. Descriptive Results

**Figure 2:**
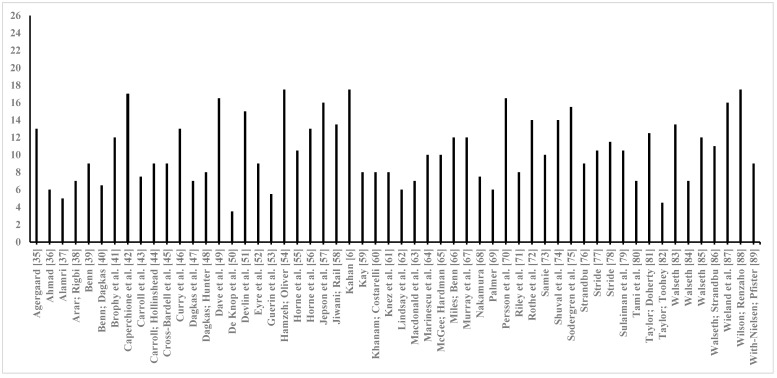
Number of COREQ items (i.e., quality scores) reported per study (*n* = 56).

Quality score values across the 56 papers [6,35,36,37,38,39,40,41,42,43,44,45,46,47,48,49,50,51,52,53,54,55,56,57,58,59,60,61,62,63,64,65,66,67,68,69,70,71,72,73,74,75,76,77,78,79,80,81,82,83,84,85,86,87,88,89] averaged 10.54 (i.e., 40.5% of the maximum score of 26) ± 3.74 (Figure 2). The median score—used to differentiate low vs. high quality papers—was 10 (i.e., 38.5% of maximum). Scores ranged from a low of 3.5 (13.5%) [50] to a maximum of 17.5 (67.3%) [6,54,88] (Figure 2).

**Figure 3:**
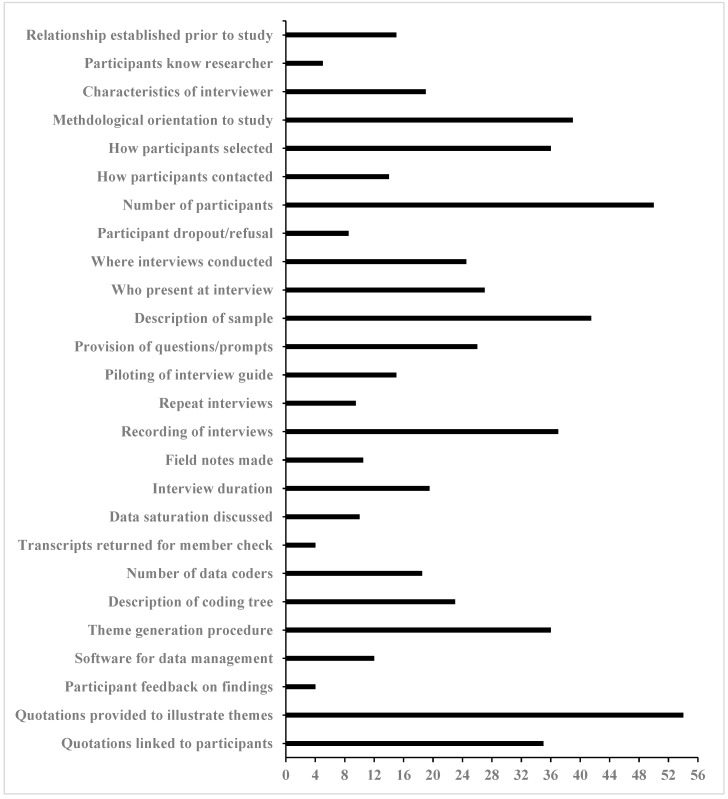
Number of studies reporting individual COREQ item (*n* = 56).

Scores across the 26 COREQ items averaged 22.83 (i.e., 40.8% of the maximum score of 56 papers) ± 14.05 (Figure 3). Eight COREQ items’ scores were lower than 14 (i.e., 25% or less of the studies reported the item). Items, with their specific wording, are included in ascending order: (1) Did participants provide feedback about the findings? (4, 7.1%); (2) Were transcripts returned for comment or correction? (4, 7.1%); (3) What did the participants know about the researcher? (5, 8.9%); (4) How many participants refused to participate or dropped out and what were reasons? (8.5, 15.2%); (5) Were repeat interviews carried out and if so, how many? (9.5, 17.0%); (6) Was data saturation discussed? (10, 17.9%); (7) Were field notes made during and/or after the interview? (10.5, 18.8%); and (8) What software, if applicable, was used to manage the data? (12, 21.4%). (Paraphrased items are found in Figure 3.) In contrast, only two COREQ items’ scores were above 42 (i.e., 75% or more of the studies reported the item). Items with their specific wording included in ascending order: (1) How many participants were in the study? (50, 89.3%); and (2) Were participant quotations presented to illustrate the themes? (54, 96.4%). (Paraphrased items are found in Figure 3.)

### 3.3. Crosstabulation Results

There was no relationship between maximum length allowed for submission and paper quality (χ^2^ = 1.0, *p* = 1.0). Papers found in the PubMed database were significantly more likely to be high quality than papers found in other databases (χ^2^ = 8.59, *p* = 0.003, φ = 0.39). Specifically, papers found in the PubMed database were 5.25 (95% CI, 1.67–16.44) times more likely to be high quality than those that were not. There was no relationship between journal quality in which a paper was published and paper quality (χ^2^ = 0.35, *p* = 0.55). Newer papers (published in or after 2011) were significantly more likely to be high quality than older papers (χ^2^ = 4.94, *p* = 0.03, φ = 0.30). Specifically, newer papers were 3.52 (95% CI, 1.14−10.88) times more likely to be high quality than older papers.

## 4. Discussion

Muslim females as a group are vulnerable to physical inactivity and qualitative focus group interviews offer first-hand accounts of the physical activity barriers and facilitators they experience. Our focus was restricted to high-income non-Muslim majority, western societies with sizeable Muslim populations. Such countries may be willing and able to accommodate the cultural and religious needs of its Muslim female population as they relate to equitable access to physical activity. Therefore, qualitative research reporting needs to be sufficiently rigorous so that researchers and practitioners can accurately create/tailor physical activity programs, venues, and policies.

### 4.1. Descriptive Findings

Based on quality scores representing the sum of 26 COREQ items [26], the 56 studies overall demonstrated low quality (i.e., 40.5% of maximum score of 26). Even the highest scoring papers reported only two-thirds of the 26 items. Meanwhile, when examining the 26 COREQ items separately, individual items averaged only 41% of papers reporting them. Moreover, for eight items less than 25% of papers reported them. These findings by themselves do not necessarily imply the research itself was of poor quality but indicate that they were published with insufficient reporting of multiple domains’ items found on the COREQ. Of particular concern are low-scoring items associated with COREQ domains 2 (study design) and 3 (analysis and findings), which may hinder replication and obfuscate interpretation. For example, only 15% of studies reported how many participants refused to participate or dropped out of focus group interviews and offered reasons (COREQ domain 2 item). Were this information reported, readers could decide if focus group interviewees truly represented the target population and potentially preventively address reasons for non-participation in future studies of their own. As a second example, only 7% of studies reported whether participants provided feedback on findings (COREQ domain 3 item). Thus, readers are left not knowing whether participants were afforded an opportunity to verify researchers’ interpretations, which would potentially offer additional layers of member checking and triangulation to enhance trustworthiness [90]. 

Some researchers deem the COREQ reductionist and proscriptive [91], while others maintain that it allows peer reviewers and journal editors to make systematic, informed decisions about manuscript quality [92]. Some journals (e.g., *Journal of Public Health*) and groups of journals (e.g., BMC) suggest or require that manuscript submissions follow COREQ reporting guidelines and may even ask authors to submit a completed checklist identifying where in the manuscript each item is addressed. Ultimately, it is the responsibility of journal editors and reviewers to ensure that these stipulations are met. Given the proliferation of open-access and online journals that are not bound to printed page limits due to fiscal considerations, and the availability of data repositories, it is reasonable to request a completed COREQ checklist be included as an appendix or link in accepted manuscripts employing focus groups.

### 4.2. Crosstabulation Findings

Pitney and Parker [20] suggested that page length and/or word limits imposed by journals may limit the scope of qualitative research article content such that thorough and transparent reporting might be compromised. No association, however, was found between article length limits imposed by journals and quality scores. Several studies have found that qualitative research in the medical and health sciences is typically published in lower impact factor clinical journals [31,32]. Conversely, another study found qualitative health care research was published in general medical/health journals with high impact factors [93]. No association, however, was found between quality score (low vs. high) and journal standing based on impact factor (Scimago quartile 1 vs. other quartiles). 

High quality scores were independently associated with an article being listed in the PubMed database vs. not and being published in 2011 and later vs. before. Journals not indexed in PubMed (e.g., *Sport, Education & Society*) typically possessed a sociological approach to inquiry independent of biomedical journal conventions that de-stressed conformity to reporting guidelines. Authors submitting to such journals may not have been aware of COREQ or other systematic reporting guidelines and instead written their manuscripts to comply with less stringent instructions to authors for reporting qualitative studies. Editors and reviewers of journals not indexed in PubMed may also be unaware of the COREQ, wish to offer greater flexibility in reporting given the diversity of qualitative methodologies, or both. 

Finding that high quality studies tended to be more recently published (i.e., in 2011 or later) may reflect several occurrences. First, the COREQ was published in 2007 and a lag may have existed between its publication, dissemination, and influence on subsequent studies’ reporting comprehensiveness. Indeed, according to *Google Scholar* metrics for the COREQ article [26] (accessed on 10/14/2019), of 6350 citations to it since 2007, 6290 (99.1%) citations reflected the time period from 2011 to October 2019. Second, manuscript submission and publication of qualitative research in medical and health sciences journals increased between the late 1990s and mid 2000s with increasing rates independently associated with editorial/methodological papers in journal and specific mention of qualitative research in author guidelines [30]. As this avenue of publication opened up, it is possible that the growing publication of qualitative articles spurred potential authors to submit manuscripts in ensuing years. In turn, these manuscripts were of higher quality informed by previous studies and tutorials. Meanwhile, research methods courses in schools of public health now often include qualitative research topics (e.g., methods, design, writing) as a course module or standalone course [94]. There are also growing calls that more qualitative research be conducted in sport and exercise science [95], which represents the purview of my focus on physical activity behavior. Research methods textbooks in the exercise sciences now routinely include content on qualitative research [96]. Thus, collectively, more recent publications’ higher quality scores may reflect the fruits of training and available resources in qualitative research methods.

### 4.3. Strengths and Limitations

Strengths of the study include its focus on a vulnerable group to physical inactivity that also resides in countries that may have the means and will to address barriers to physical activity. Using a validated, highly cited reporting checklist, I analyzed 56 studies—published over a 30-year time span—offering researchers and clinicians an accounting of the comprehensiveness and transparency of studies by which to judge the quality of evidence. Meanwhile, I acknowledge that the findings are limited to a specific qualitative data collection strategy and to specific behavior (physical activity), population (Muslim women), and context (high-income westernized countries). Additionally, since the conclusion of the data collection phase in June 2016, an additional unknown number of papers that would meet eligibility criteria for inclusion may have been published. Replications or extensions of this study, therefore, should account for these.

### 4.4. Implications

Despite decades of published focus group research on the topic, this study is the first to evaluate the rigor of individual studies comprising the evidence base. The review provides evidence that studies using focus groups to investigate Muslim women’s perceptions of physical activity barriers and facilitators have room to improve the thoroughness and transparency of reporting. Such exposition is important and timely because most health behavior interventions with Muslims focus on physical activity, are lay led, and are difficult to replicate because they are so poorly described [97]. I am optimistic that reporting will improve generally, as better reporting has occurred chronologically, and specifically for papers submitted to journals indexed in PubMed given their penchant for observing systematic reporting protocols. Accountability for improved reporting, however, ultimately resides with journal reviewers and editors. Meanwhile, I encourage critical appraisals of other health behaviors using the same or different qualitative techniques and with a variety of population groups in order to identify common deficiencies, and to thus strengthen the impetus for change. 

## 5. Conclusions

The body of studies using focus groups to report Muslim women’s perceptions of physical activity barriers and facilitators were of low-to-moderate quality because they omitted or incompletely reported COREQ reporting guidelines items. Even the highest quality papers of the sample only scored 67% of the maximum COREQ score. Yet more recently, published papers and those indexed in the PubMed database were more likely to be of a high quality and may reflect the dissemination and usage of the COREQ since its publication in 2007. Qualitative researchers who work in the health and medical sciences should be trained to systematically report data about their research team and reflexivity, study design, and analysis and findings; while manuscript reviewers and journal editors should hold authors accountable to reporting guidelines and standards.
